# Stabilization of the Hinge Region of Human E-selectin Enhances Binding Affinity to Ligands Under Force

**DOI:** 10.1007/s12195-021-00666-z

**Published:** 2021-02-04

**Authors:** Thong M. Cao, Michael R. King

**Affiliations:** Department of Biomedical Engineering, Vanderbilt University, Nashville, TN 37235 USA

**Keywords:** E-selectin, Conformational states, Selectin ligand binding dynamics, Molecular dynamics, Steered molecular dynamics

## Abstract

**Introduction:**

E-selectin is a member of the selectin family of cell adhesion molecules expressed on the plasma membrane of inflamed endothelium and facilitates initial leukocyte tethering and subsequent cell rolling during the early stages of the inflammatory response *via* binding to glycoproteins expressing sialyl Lewis^X^ and sialyl Lewis^A^ (sLe^X/A^). Existing crystal structures of the extracellular lectin/EGF-like domain of E-selectin complexed with sLe^X^ have revealed that E-selectin can exist in two conformation states, a low affinity (bent) conformation, and a high affinity (extended) conformation. The differentiating characteristic of the two conformations is the interdomain angle between the lectin and the EGF-like domain.

**Methods:**

Using molecular dynamics (MD) simulations we observed that in the absence of tensile force E-selectin undergoes spontaneous switching between the two conformational states at equilibrium. A single amino acid substitution at residue 2 (serine to tyrosine) on the lectin domain favors the extended conformation.

**Results:**

Steered molecular dynamics (SMD) simulations of E-selectin and PSGL-1 in conjunction with experimental cell adhesion assays show a longer binding lifetime of E-selectin (S2Y) to PSGL-1 compared to wildtype protein.

**Conclusions:**

The findings in this study advance our understanding into how the structural makeup of E-selectin allosterically influences its adhesive dynamics.

## Introduction

E-selectin belongs to a family of cell adhesion (E-, L-, and P-) molecules that are responsible for leukocyte recruitment to inflammation sites.^18^ E-selectin is a Ca^2+^-dependent lectin glycoprotein that is expressed on the plasma membrane of activated vascular endothelial cells by pro-inflammatory stimuli.^14^ Beyond its well-characterized role in leukocyte trafficking, E-selectin is also involved in integrin activation, hematopoietic stem cell homing to the bone marrow, and cancer metastasis.^4^,^15^,^25^,^26^,^35^,^42^ All selectins have a C-type lectin domain at the extracellular N-terminus containing a binding pocket that recognizes glycoproteins and glycolipids decorated with tetrasaccharide sialyl Lewis^X^ and sialyl Lewis^A^ (sLe^X/A^).^39^ Known E-selectin ligands include P-selectin glycoprotein ligand-1 (PSGL1), CD43 and CD44 expressed on various human leukocyte subpopulations, and E-selectin ligand-1 (ESL-1) present on mouse myeloid cells.^8^,^12^,^17^ Connecting to the lectin domain is an epidermal-growth-factor (EGF-like) domain, followed by varying units of short consensus repeats (SCRs), a transmembrane portion and ending with a cytoplasmic tail.^14^ Structural studies of selectins suggest that the molecules can exist in two conformational states: a low affinity (bent) conformation and a high affinity (extended) conformation.^11^,^23^,^31^,^37^ The defining characteristic differentiating the two conformational states is the interdomain angle of the hinge region, the interdomain region connecting the lectin to the EGF-like domain. The hinge region of selectin is of the focus in this study because of its allosteric modulation of selectin binding mechanics.^28^,^33^ Here we show a single amino acid substitution at the hinge region can alter the overall macromolecular characteristics of E-selectin resulting in altered binding kinetics to ligands. The data supporting this observation will be discussed in this article.

Effective leukocyte trafficking in the vascular microenvironment under physiological shear flow requires that selectins are able to bind to ligands very quickly by having a fast on-rate. In addition, the subsequent selectin/ligand complex must able to withstand the pressure of the tensile stress. Selectin binding exhibits what is known as “catch-bond” behavior in which receptor/ligand bond formation requires a minimum threshold level of shear.^7^,^21^,^41^ The bond is further strengthened as the tensile force increases until the force becomes too great, and the bond becomes an ordinary “slip-bond”. Previous studies have put forth multiple models linking the structural architecture of selectin’s conformational states to their catch-bond behaviors. For instance, in the “sliding-rebinding” model described by Lou *et al*., it is postulated that the flexibility of the hinge region in L-selectin allows it to adopt two different conformations: a low affinity (bent) conformation in the absence of force and a high affinity (extended) conformation in the presence of force.^19^,^20^ In the extended conformation, the binding surface is aligned with the direction of the force which allows bound ligands to slide along the binding interface and continuously rebuild or form new interactions, causing a high affinity conformation. Selectin crystal structures also revealed significant structural displacement at the binding interface when the molecule adopts different conformational states, which suggests an allosteric relationship between the hinge region and the binding interface.^40^ However, the “sliding-rebinding” model does not address this allosteric relationship. Alternative theories have been proposed linking the force-induced extension of selectin molecules and allosteric changes in the ligand binding interface.^38^,^40^ In the “prybar” model, residue 1W of the lectin domain and the EGF-like domain of L-selectin together form an L-shaped lever that is held together by a network of hydrogen bonds when the molecule is in the bent conformation. When force is applied and L-selectin transitions to the extended conformation, the lever dislodges the relatively large and hydrophobic residue, 1W, which causes a cascade of residue displacements resulting in the allosteric change of the binding interface to a high-affinity conformation.

In different species, the same selectin molecules share a high degree of amino acid sequence homology: ~72 and ~60% of amino acid sequence are conserved for the lectin and EGF-like domain, respectively.^14^ The high degree of inter-species sequence homology enables mouse E-selectin to readily cross-react to human ligands and vice-versa.^22^,^34^ More interestingly, mouse E-selectin exhibits significantly higher affinity to human ligands than human E-selectin. This observation can be explained in part by molecular dynamics (MD) simulations that found mouse E-selectin to have a greater interdomain angle than its human counterpart, suggesting that mouse E-selectin favors the high-affinity extended conformation.^34^ However, individual amino acid variances of E-selectin between human and mouse species and their influence on ligand binding kinetics remain largely unexplored. The goal of this study was to gain new insight into E-selectin conformational states, their corresponding ligand binding characteristics, and how strategic structural modifications of E-selectin can modulate its macromolecular behavior. Using MD simulations, we predicted possible E-selectin structural transitions between conformational states and their effects on the binding interface. We also looked at the network of non-covalent interactions distributed over the hinge region associated with these conformational changes. Next, we introduced a key point mutation at the hinge region and examined the dynamic changes over time on E-selectin/PSGL-1 complex in the presence of a pulling force that was applied to induce unbinding. In conjunction with computational modelling, experimental cell rolling assays were used to substantiate our MD results.

## Results

### E-Selectin Spontaneously Transitions Between the Bent and Extended Conformations at Equilibrium

**Figure 1:**
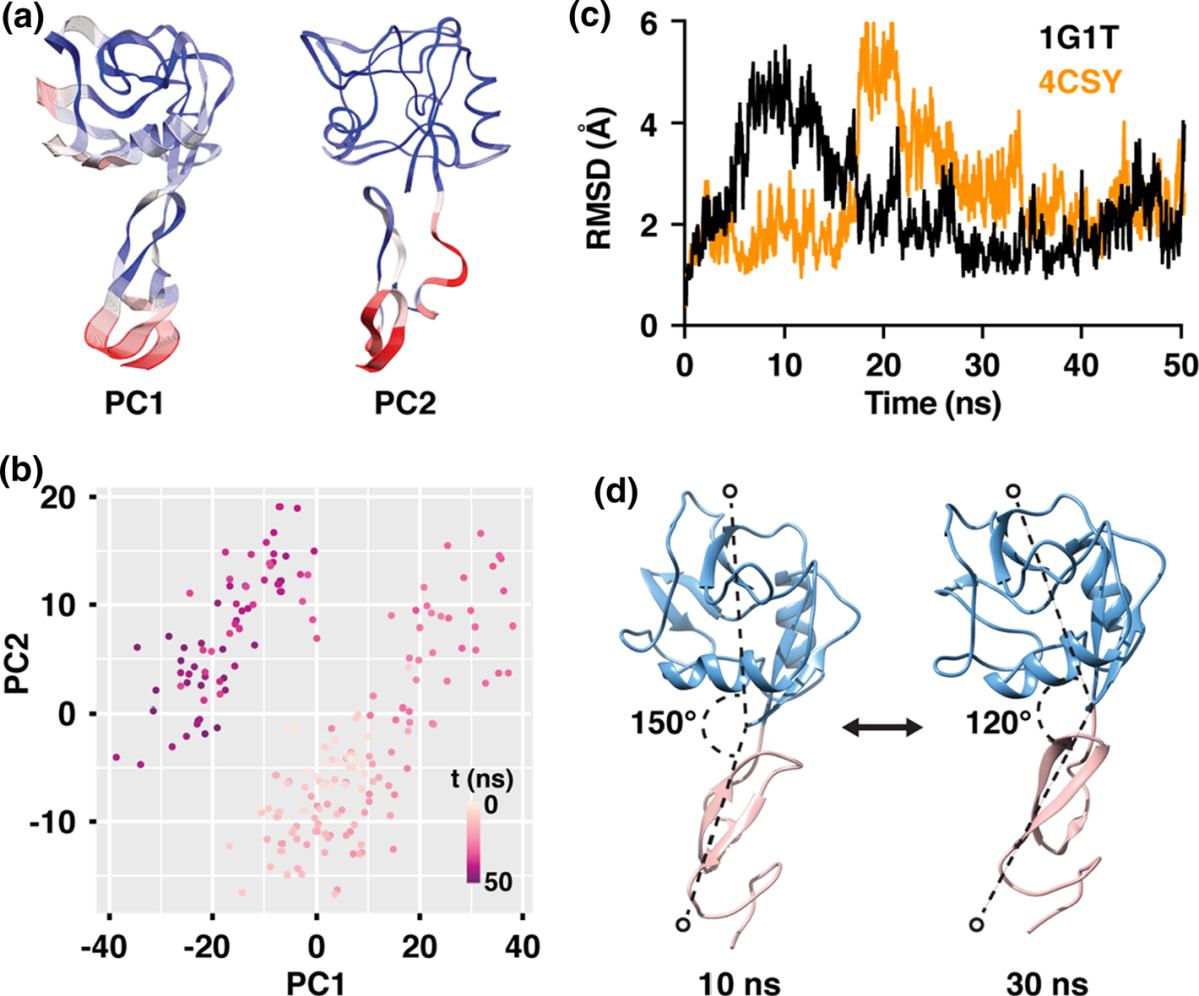
E-selectin spontaneously transitions from bent to extended conformational state and back again. (**a**) MD trajectory representations of E-selectin during the least displacement and the highest displacement, respectively. (**b**) PC1 vs PC2 shows the predicted transitioning path of E-selectin from bent, to extended, and back to bent. (**c**) RMSDs of MD simulations as a function of time. (**d**) Angle measurement of the interdomain hinge region as a function of time. (**e**) Ribbon representations of E-selectin and the measured angle at *t* = 10 ns and *t* = 30 ns. (*Blue* lectin domain, *Pink* EGF -like domain).

Existing structural data suggest that selectins adopt two conformational states: a low affinity (bent), and a high affinity (extended) conformation. Furthermore, MD simulations predicted that L-selectin can undergo conformation switching in the absence of force.^30^ To determine if E-selectin exhibits spontaneous conformational change between the two conformational states in the absence of force, we simulated the molecule, as previously described,^2^ using the published crystal structures of the two known conformational states (bent - 1G1T, extended - 4CSY) as starting coordinates. Principal component analysis (PCA), a dimensionality reduction technique,^9^,^43^ was used to transform the original high-dimensional representation of protein motion in MD simulations into a lower dimension representation that reveals the dominant structural dynamics of proteins. The vibrational fluctuation of the molecules over a period of 50 ns of simulation time was reduced to two Principal Components (PC 1, PC 2), which attributed for over 78% of total protein movement. The greatest fluctuation was observed in the EGF-like domain (residues 129-) (Fig. 1a). In addition, the two domains moved in opposite directions of one another. In the lectin domain, the flexible region adjacent to the hinge region, and the flexible loop (residues 83–89) at the binding pocket also showed a high degree of fluctuation (Fig. 1a). Tracing the trajectory of the structural displacement of the simulation of 4CSY (extended) we observed that the molecule spontaneously transitioned from the bent to extended conformation and back again (Fig. 1b). RMSD traces of the simulations of either 4CSY (extended conformation) or 1G1T (bent conformation) showed conformational changes occurring at 10 and 20 ns, respectively (Fig. 1c). These conformational changes correlated with the opening and closing of the hinge region as indicated by the opening and closing of the interdomain angle (Fig. 1d).

### A Dynamic Network of Hydrogen Bonds at the Interdomain Hinge Region Stabilizes the Conformational States

**Figure 2:**
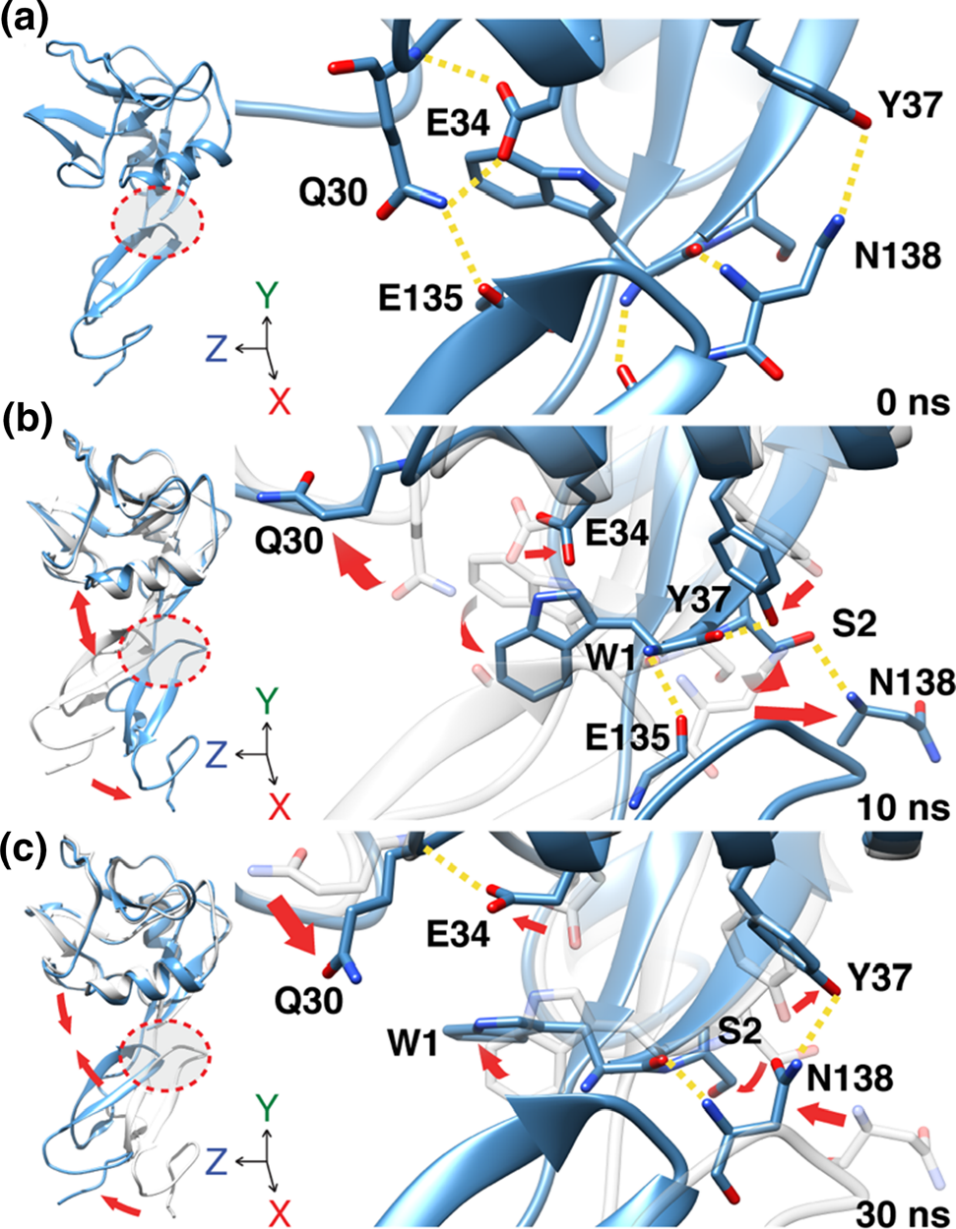
Dynamic hydrogen network stabilizes the interdomain hinge region. Representative illustrations of human E-selectin MD simulations at indicated time points showing the formation and breakage of hydrogen bond network as the protein transitions from the bent to the extended conformation and back again at the interdomain region. Yellow dashed lines represent hydrogen bonds; Red arrows indicate trajectories of indicated residues.

A closer examination of the flexible interdomain hinge region during the oscillation between the bent and extended conformational states revealed a dynamic hydrogen bond network. Figure 2 shows three representative simulation time points of an MD simulation of E-selectin transitioning from a bent to an extended conformation and back again. Looking directly down the X-axis of E-selectin protein, there existed a network of hydrogen bonds (H-bond) that stabilizes the interdomain region on both sides of the molecule. At time = 0, when the molecule is in the bent conformation and the interdomain angle is at its minimum, residue Q30 of the lectin domain forms a hydrogen bond with E135 of the EGF-like domain. This stabilizes E-selectin in the bent conformation (Fig. 2a). Residue E34 formed an H-bond to residue Q30, thus keeping the residue facing in close proximity to residue E135. This further promotes its interaction with residue E135. On the opposite side of the molecule, residue N138 of the EGF-like domain exhibited multiple hydrogen bonds with residues on the lectin domain: residue Y37 and the carboxyl oxygen of residue W1. This network of hydrogen bonds between the lectin and the EGF-like domain collectively maintains E-selectin in a bent conformation.

As E-selectin shifted towards the extended conformation and the interdomain angle became larger (Fig. 2b), residue E34 shifted to the right and away from Q30 and no longer hydrogen bonded to residue Q30, allowing residue Q30 to rotate upward and away from the EGF-like domain. Thus, residue Q30 no longer interacted with residue E135, which also shifted to the right. As the loop region was moving away, residue W1 swung downward and towards the right which then hydrogen bonded with residue E135 (shifted far to the right) and residue Y37. Most noticeably, residue S2 flipped its orientation towards the front and then formed a hydrogen bond with residue N138. The disproportional distribution of hydrogen bonds present here favors the extended conformation.

At *t* = 30 ns, E-selectin reverted back to the bent conformation (Fig. 2c). On the left side of the interdomain region, residue Q30 re-rotated back downward and towards the front, bringing it, again, closer to EGF-like domain. Residue W1 swung upward and to the left.

The dynamic breaking and formation of hydrogen bonds and the amino acid residues involved as E-selectin transitions between conformational states suggest that these amino acids, to varying degrees, influence the macromolecular structure of E-selectin.

### A Substitution of Residue 2 (S2Y) Eliminates Spontaneous Transitioning Between the Two Conformational States

**Figure 3:**
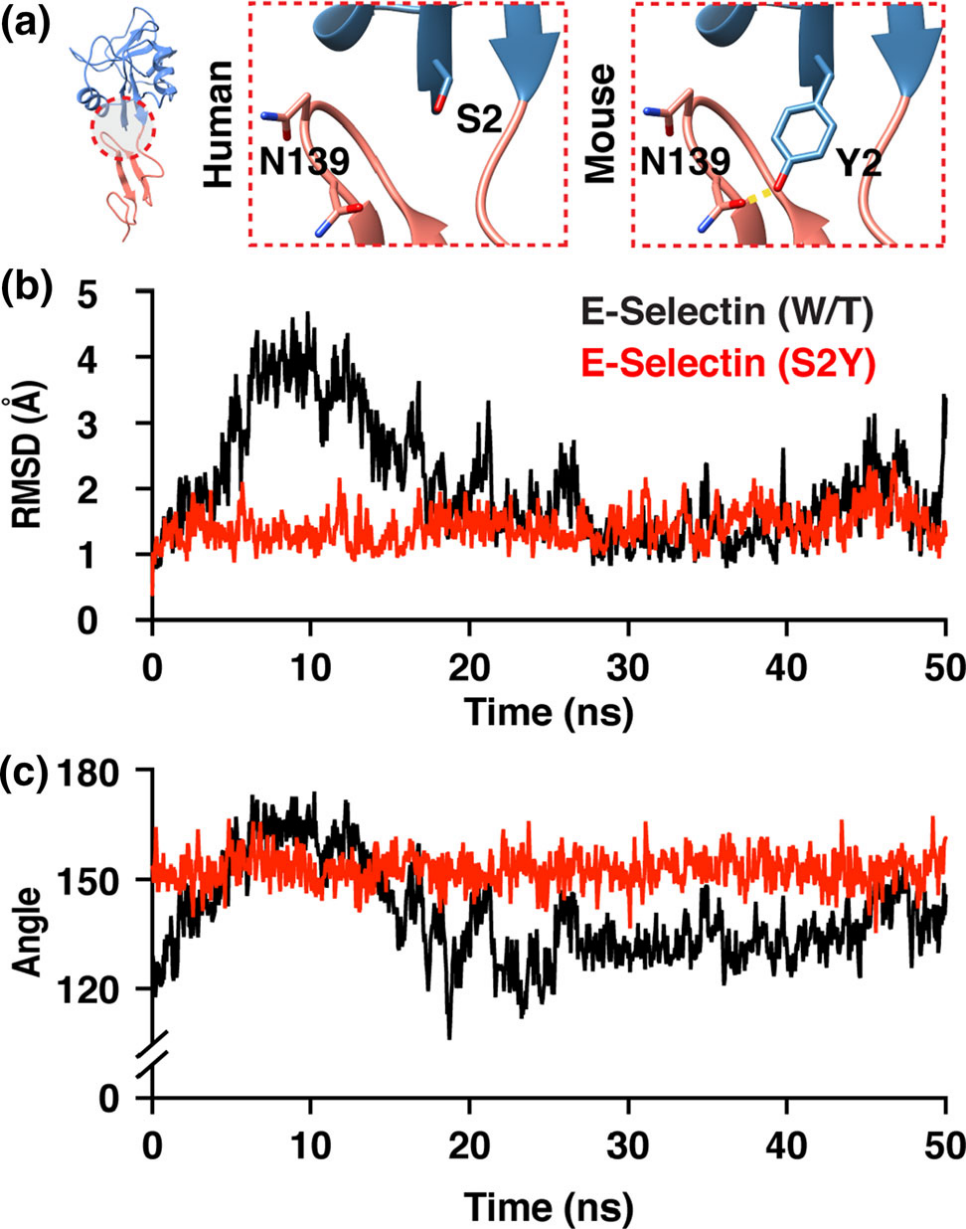
Point mutation of residue 2 from Serine to Tyrosine eliminates the spontaneous transition between the two conformational states.

There exist 20 amino acid differences within the lectin domain between human and mouse E-selectin.^34^ Of these 20 variations, only residue number 2 (S2) of the lectin domain is located in the hinge domain and is involved in noncovalent interaction with residues in the EGF-like domain as pointed out in Fig. 2. Serine is a smaller amino acid, and its side chain consists of a hydroxyl group that can noncovalently react with other molecules. On the mouse E-selectin molecule, the serine residue is replaced with a much larger tyrosine residue that also has a distal hydroxyl group on its sidechain which can readily form H-bonds with asparagine residues (N138 and N139) on the EGF-like domain (Fig. 3a). Based on the simulation results suggesting that interactions between residue 2 and other residues in the EGF-like domain favor the extended conformation, we postulated that a S2Y point mutation of the human E-selectin molecule would cause the molecule to favor the high affinity extended conformation over the lower affinity bent conformation. To test this hypothesis, we simulated human E-selectin (S2Y) in the similar fashion to the wildtype counterpart, E-selectin (W/T). The S2Y point mutation effectively abolished any spontaneous transitioning of E-selectin between conformational states, as evident by the RMSD trace and the interdomain angle measurements (Figs. 3b and 3c). The E-selectin (S2Y) molecule remained in the high affinity extended conformation throughout the entire simulation.

### High Affinity Extended Conformation Allosterically Forces the Binding Pocket to Also Adopt a High-Affinity Conformation in E-Selectin

**Figure 4:**
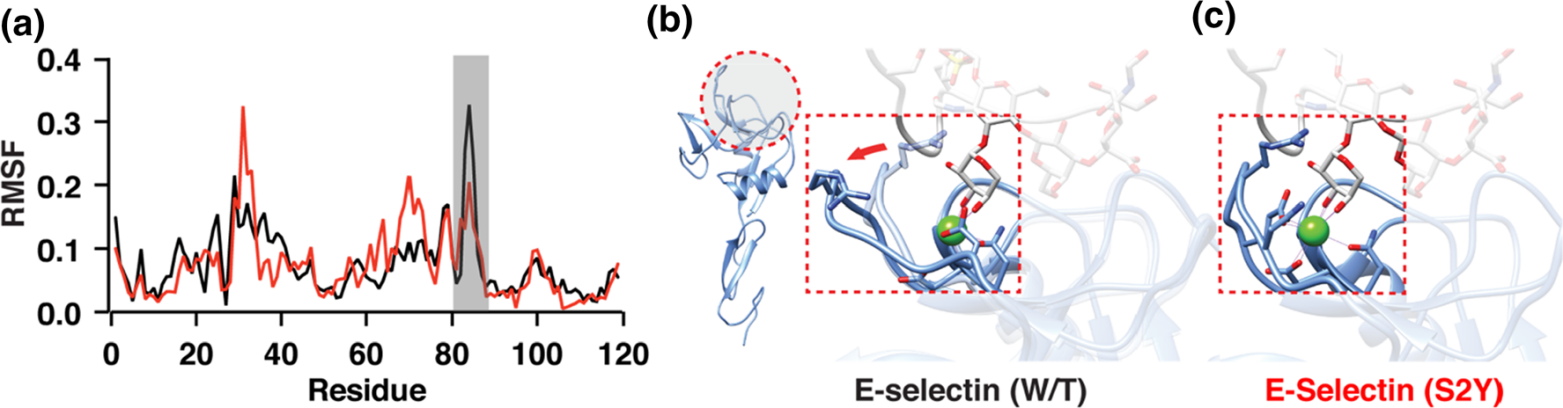
High affinity extended conformation allosterically forces the binding pocket to also adopt a high-affinity conformation in E-selectin. (**a**) RMSF per residue of the lectin domain of wildtype E-selectin (W/T) and variant E-selectin (S2Y). Grey rectangle highlights the flexible loop containing residues 83-85 of the binding pocket. (**b**) Schematics of flexible loop outlining the reduction of fluctuation in E-selectin (WT) compared to E-selectin (S2Y).

Conformational changes in the hinge region of selectins can allosterically induce conformational changes to the binding region.^5^,^30^ We compared the structural changes occurring in our simulations between the two E-selectin variants. RMSF trace over the simulation time revealed significantly higher residue fluctuations at the flexible loop containing residues 81–85 for E-selectin (W/T) compared to E-selectin (S2Y) (Fig. 4a). This flexible loop which is located at the binding pocket and its amino acids are involved in ligand binding.^14^ For the wildtype E-selectin, the loop alternated between high-affinity and low-affinity conformation which reduced the overall exposure time of these amino acid residues to the bound ligand (Fig. 4b). Conversely, for simulations involving E-selectin (S2Y), the same flexible loop remained for almost the entire simulation time in the high-conformation state.

### E-Selectin (S2Y) Exhibits Higher Binding Affinity to Its Ligand Than Wildtype E-Selectin

**Figure 5:**
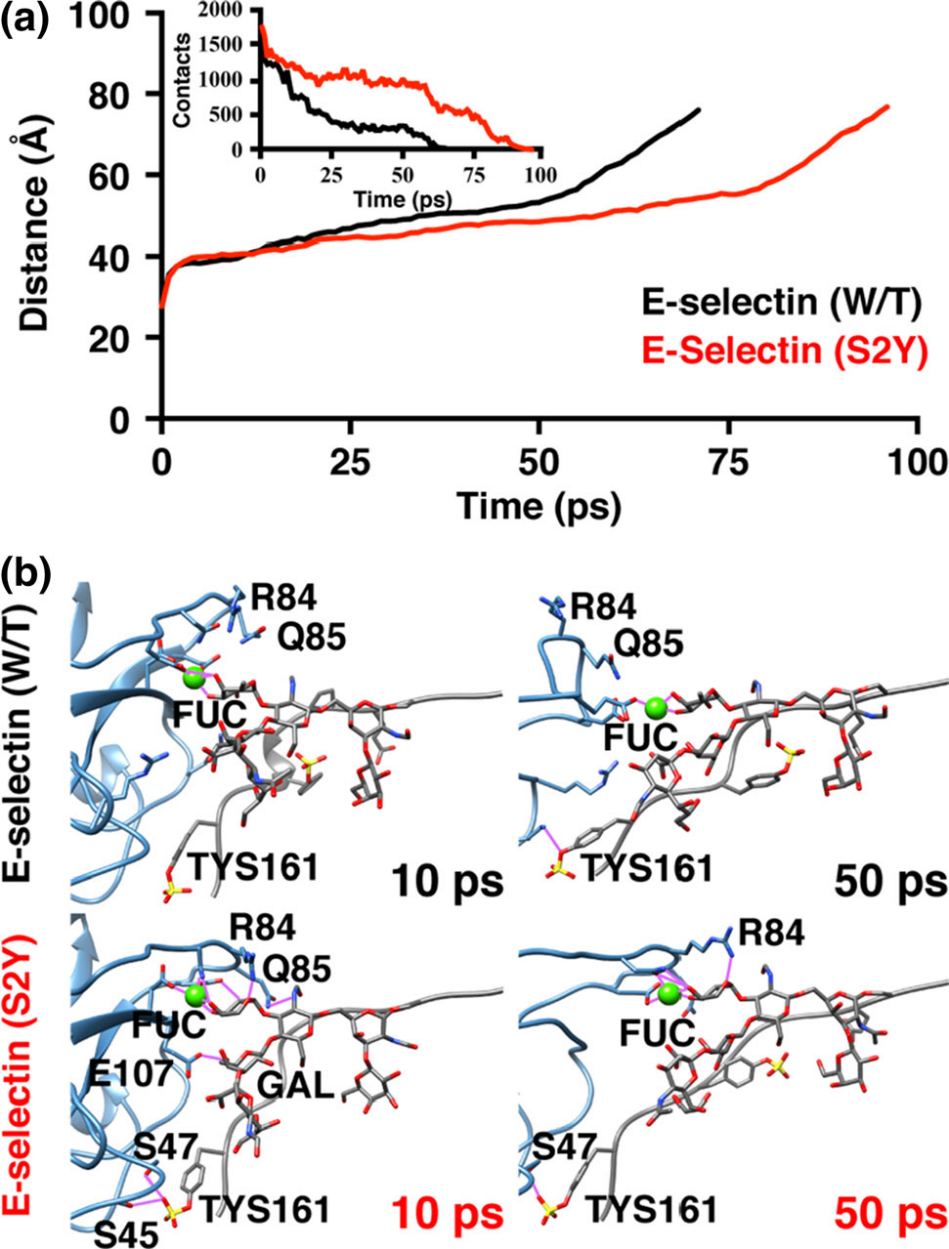
SMD simulations show PSGL-1 has higher affinity to E-Selectin (S2Y) than E-Selectin (W/T). (**a**) SMD simulation showing the dissociation time (in simulation time) of PSGL-1 from E-Selectin. (**b**) Representative illustrations of human E-selectin interaction with PSGL-1 as it is being pulled away from E-Selectin at the indicated time points. E-selectin is blue; PSGL-1 is grey. Purple lines are representative of inter-molecular interactions.

To test our hypothesis that a single amino acid substitution (serine to tyrosine) at residue 2 of E-selectin would allosterically extend the binding lifetime of E-selectin to PSGL-1, we used steered molecular dynamics (SMD) simulations to manually dissociate PSGL-1 away from E-selectin as previously described.^2^,^34^ Figure 5 illustrates the amount of simulation time of a representative simulation necessary before PSGL-1 is completely dissociated from E-selectin (W/T and S2Y), as defined by the distance at which non-covalent intermolecular interactions between the two molecules are no longer observed. The SMD simulation showed that PSGL-1 completely dissociated from E-selectin (W/T) significantly faster than with E-selectin (S2Y) at approximately 70 ps and 95 ps, respectively (Fig. 5a). Four SMD simulations were performed with different initial velocities for E-selectin (W/T or S2Y)/PSGL-1 complex. The average dissociation times were 52.5 ± 14.3 ps and 71.3 ± 23.5 ps for wildtype E-selectin and E-selectin (S2Y), respectively. The longer binding time between PSGL-1 and E-selectin (S2Y) was likely the result of the flexible loop at the binding pocket remaining in close proximity to PSGL-1, thus allowing residues R84 and Q85 to experience prolonged interactions with the fucose residue on PSGL-1. At 10 ps after the initial pulling of PSGL-1 away from E-selectin, the flexible loop containing residues R84 and Q85 in E-selectin (WT) already starting to fold away from the binding pocket resulting in residues R84 and Q85 to stop interacting with PSGL-1 (Fig. 5b, top left panel). Conversely, the same flexible loop in E-selectin (S2Y) was still facing into the binding pocket, allowing residues R84 and Q85 to maintain multiple contacts with the fucose molecule on PSGL-1 (Fig. 5b, bottom left panel). Furthermore, PSGL-1 also maintained multiple interactions with E-selectin (S2Y) outside of the binding pocket including: residue E107 with the galactose, and serine residues (S45, S47) with the sulfated tyrosine (TYS161) on PSGL-1. These interactions were absent in the simulation involving E-selectin (W/T). At 50 ps, the flexible loop inside the binding pocket of E-selectin (W/T) had completely folded away from PSGL-1, further weakening the interaction between the two molecules (Fig. 4b, top right panel). This is evident from the increase in dissociation distance rate that followed (Fig. 5a). Comparing to its wildtype counterpart, the E-selectin (S2Y) still maintained multiple interactions with PSGL-1 in the binding pocket, including residue R84 interacting with one of the oxygen species of the fucose molecule on PSGL-1 (Fig. 4b, bottom right panel).

**Figure 6:**
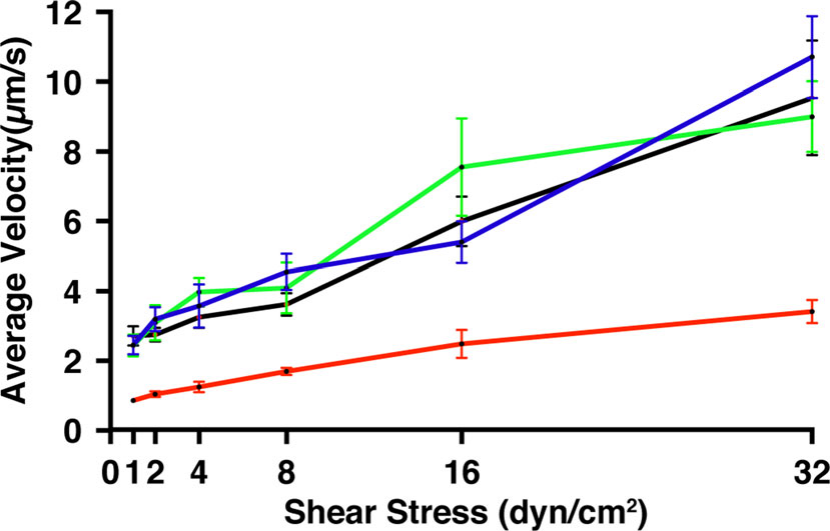
E-selectin (S2Y) has increased adhesiveness to ligands compared to the wildtype. The average rolling velocity of model cell line, KG-1a, ligands rolling on microtubes coated E-selectin-Fc chimeric protein (filled black square wildtype, filled red square S2Y, filled green square S2W, filled blue square S2C; *n* = 3).

To further corroborate the SMD simulation results, we experimentally assessed the effect of the point mutation (S2Y) on E-selectin’s ligand affinity. We performed a cell rolling assay using model cell line KG-1a, in microtubes coated with the indicated E-selectin/Fc chimeric molecules and under a range of physiological shear stresses as previously described.^2^,^13^ KG-1a is a leukemic cell line expressing abundant selectin ligands on its surface, and is a commonly used model cell line in rolling assays for studies involving selectin ligand binding mechanics.^24^,^32^ Human E-selectin/Fc chimeric protein was created and purified from HEK 293 cells. Variants of wildtype including E-selectin (S2Y, S2W, and S2C) were also created and purified to assess the allosteric effect of the hinge on E-selectin binding behavior. The average rolling velocities of KG-1a cells were significantly lower on surfaces coated with E-selectin (S2Y) at all levels of shear stress compared to E-selectin (W/T) (Fig. 6). This is indicative of E-selectin (S2Y)’s enhanced ligand binding compared to the wildtype. We expected the capability of the tyrosine residue to hydrogen bond to asparagine residues in the EFG-like domain would shift the equilibrium in favor of the high affinity extended conformation. Therefore, we also examined the rolling velocity of KG-1a cells on surfaces coated with E-selectin (S2W). Tryptophan (W) is similar to tyrosine in structure, as it is also a bulky amino acid that has an aromatic ring. However, it cannot hydrogen bond to residues 138 and 139 because it lacks the functional OH group. As a result, the rolling velocity profile of KG-1a was observed to be very similar to that of the wildtype. We also looked at the rolling profile of cells on surfaces coated with E-Selectin (S2C). Cysteine has a similar structure to serine but has a sulfur in place of an oxygen atom at the gamma location. Not surprisingly, the rolling profile of KG-1a on this surface was also similar to that on the wildtype. The experimental data support our theory that a S2Y substitution favors the high affinity extended conformation in E-selectin.

## Discussion

The two-state kinetic model of selectin’s catch bond behavior postulates that, in the absence of force, selectins may exist in two interchanging conformational states at equilibrium.^1^,^6^ When tensile force is applied, selectins favor the high affinity, extended conformation. This theory is supported by structural studies of E- and P-selectin and computational modelling of L-selectin.^7^,^11^,^20^,^21^,^23^,^28^,^31^,^33^,^41^ Here, our MD simulation data of E-selectin also predict that the molecule does indeed follow the two-state model, as observed by the molecule’s spontaneous transitioning between the bent and extended conformational states (Fig. 1). Furthermore, we observed conformational change in 3 out of the 4 simulation runs we performed for each starting coordinate (bent and extended). An observed 75% occurrence rate in our simulations is higher than that seen by Lou *et al*. (~30%) in their MD simulation of L-selectin. The frequency increase may be explained by the extended simulation time of 50 ns in our simulations, compared to 6 ns in the previous study.^20^ In a more recent study by Preston *et al*. using small angle X-ray scattering (SAXS) measurement, they found that in the absence of force, ligand binding to E-selectin alone can allosterically shift the equilibrium to favor the high-affinity, extended conformation.^31^ This observation is in contrast to earlier assertions that tensile force is required to shift the conformational equilibrium towards the extended conformation.^38^ More importantly, these seemingly contradicting observations emphasize that while our understanding of selectin mechanochemistry is expanding, it is still incomplete. Although it was not explored in this study, it would be interesting in future studies to use MD techniques such as docking to determine if ligand binding alone can trigger an allosteric structural change from the bent to extended conformation in selectins.

Interaction between the lectin and EGF-like domain of selectins is limited in the number of contacts, as observed in published crystal structures and the results presented here (13, 14, Fig. 2). The small number of interactions between the two domains supports the theory that the hinge region of selectins is inherently flexible, suggesting that thermodynamically the molecule can readily transition between conformational states at equilibrium. Moreover, by modulating the degree of flexibility of the hinge region, one may influence the conformational equilibrium of selectin. This idea was previously explored in P-selectin which showed that locking P-selectin in the extended conformation can increase its adhesiveness to ligands under hydrodynamic force.^30^ In addition, others have introduced an N138G substitution on the EGF domain of L-selectin, and showed that replacing a bulky asparagine residue with a smaller glycine residue, can stabilize the high affinity extended conformation of L-selectin.^20^,^30^,^38^ Here we investigated the stabilizing effect of single amino acid substitution at residue 2 (S2Y) on the lectin domain of E-selectin. While human and mouse E-selectin share considerable sequence homology, there exist 20 amino acid variances in the lectin domain that may, to varying degrees, contribute to enhanced affinity seen in mouse E-selectin to human ligands.^34^ Whereas residue 2 in human E-selectin is serine, mouse E-selectin has a relatively bulkier amino acid tyrosine at residue 2. A serine to tyrosine substitution of human E-selectin led to significant decrease in rolling velocity of KG-1a cells on surface coated with E-selectin across a range of shear stresses, an indication of stronger ligand affinity (Fig. 6). The bulky aromatic ring of tyrosine forces the amino acid to adopt the most energetically favorable orientation, facing away from the lectin domain and with its distal OH group pointing at residues N138 and N139.^3^ In the bent conformation, neither serine nor tyrosine interacts with residues in the EGF domain. However, when E-selectin is in the extended conformation, only the larger tyrosine residue is able to make sidechain hydrogen bonds with residues 138 and 139 on the EGF domain at high frequency. We hypothesized that during one of E-selectin spontaneously transition from bent to extended conformation, the S2Y substitution affords more opportunity of H-bond formation with residues 138 and 139 on the EGF domain. As a result, the conformation equilibrium favors the extended conformation. The absence of conformational change in simulations for E-selectin (S2Y) using the extended conformation as starting coordinates supports this hypothesis (Fig. 3). Furthermore, SMD simulations of E-selectin/PSGL-1 complex showed extended binding for E-selectin (S2Y) compared to the wildtype. Collectively these results suggest the importance of allosteric dependence of the hinge region on the binding mechanics of selectins. A better understanding of selectin structure and its influence on ligand binding can be applicable to other receptor/ligand complexes that exhibit the similar catch-bond behavior.

It is important to note that we did not observe in our simulations of E-selectin (S2Y) with the bent conformation as starting coordinates the molecule spontaneously transition to the extended conformation and remain so. This was not completely unexpected given the limitations of MD simulations. MD simulation time scale represents only a tiny fraction of the biological time scale. Therefore, extended simulation time covering multiple conformational switching would be necessary to produce a truly comprehensive representation of protein biomechanics at the biological time scales. Ultimately, MD simulation time is limited by computing power. Moreover, current molecular mechanics force fields used by many MD simulations do not fully capture the directional dependence of hydrogen bonds.^27^,^36^ This makes modelling selectins, with their extensive and complex hydrogen networks a continuing challenge.

## Methods

### Molecular Dynamics (MD) Simulation

The bent and extended crystal structures of E-selectin (1G1T, 4CSY^31^,^37^), were obtained from the Protein Data Bank for use as starting atomic coordinates. Free-dynamics and steered molecular dynamics (SMD) simulations were performed using the YASARA (http://yasara.org) package of MD programs with the YAMBER3 self-parameterizing force field. For all simulations, the temperature and pressure were held constant at 300 K and 1 atm, respectively. Periodic boundary conditions, the particle mesh Ewald method for electrostatic interactions, and the recommended 7.86 Å force cutoff for long-range interactions were also used. Predicted structures of PSGL-1 bound to E-selectin were obtained by aligning E-selectin (1G1T, 4CSY) crystal structures on the P-selectin/PSGL-1 complex (1G1S, subunit a) *via* the MUSTANG algorithm. All simulations were run at least four times with different starting velocities.

Principle Component Analysis (PCA) of MD simulations was performed with Bio3D, and R software packaged developed by the Grant lab.^10^ Cartoon renditions of E-selectin was created with UCSF Chimera, an extensible molecular modelling system, developed by the UCSF Resource for Biocomputing, Visualization, and Informatics.^29^

### Cell Lines and Cell Culture

The acute myeloid leukemic KG-1a cell line (ATCC number CCL-264.1) was purchased from ATCC (Manassas, VA) and cultured in RPMI 1640 media supplemented with 2 mM L-glutamine, 25 mM HEPES, 10% (v/v) FBS, and 100 U/mL penicillin-streptomycin at 37C and 5% CO_2_. Cultured cells were regularly test for mycoplasma using Universal Mycoplasma Detection Kit (ATCC 30-1012K).

### Microtube Functionalization

The microtubes were functionalized using methods, as previously described.^16^,^32^ Briefly, micro-renathane (MRE) tubes (300 mm i.d.; Braintree Scientific, Braintree, MA) were sterilized with 75% ethanol for 15 min. After three washes with phosphate buffer saline (PBS) (Ca^2+^ and Mn^2+^ free), the microtubes were incubated with Protein G (2 *μ*g/mL PBS) for 1 h. Next, the luminal surface was functionalized with chimeric human E-selectin (W/T, S2Y, S2W, S2C) at 5 *μ*g/mL for 2 h. The microtubes were then incubated with dry milk powder (5% w/v) in PBS for 1 h to minimize nonspecific adhesion. For control experiments, microtubes were prepared as indicated above except that the adhesion molecule was replaced with bovine serum albumin (BSA).

### Cell Adhesion Assay

Cell rolling experiments were conducted using methods as previously described.^2^ KG-1a cells were suspended in flow buffer (PBS supplemented with 2 mmol Ca^2+^) at the concentration of 10^6^ cells/mL. Cell suspension was perfused through functionalized microtubes using a syringe pump at specified wall shear stresses. Videos were recorded for 1 min at 5–10 random locations along the length of the microtube after 5 min of perfusion for each shear stress.

### Data Acquisition

Rolling cells were recorded using a microscope-linked Hitachi CCD camera KP-M1AN (Hitachi, Japan) and a Sony DVD Recorder DVO- 1000MD (San Diego, CA). Rolling velocity was determined using ImageJ (U.S. National Institutes of Health, Bethesda, MD). Rolling cells were defined as a cell translating along the surface for >2 s at a velocity <50% of the free stream velocity of a noninteracting cell. The average rolling velocity was calculated from at least 30 rolling cells. Cell rolling experiment was done in triplicates for statistical analysis. Symmetric unpaired *t* tests were used to analyze results.
